# Predicting Long-Term Sickness Absence and Identifying Subgroups Among Individuals Without an Employment Contract

**DOI:** 10.1007/s10926-020-09874-2

**Published:** 2020-02-06

**Authors:** Ilse Louwerse, H. Jolanda van Rijssen, Maaike A. Huysmans, Allard J. van der Beek, Johannes R. Anema

**Affiliations:** 1 Department of Public and Occupational Health, Amsterdam Public Health Research Institute, Amsterdam UMC, Vrije Universiteit Amsterdam, Van der Boechorststraat 7, NL, 1081 BT Amsterdam, The Netherlands; 2 Dutch Institute of Employee Benefit Schemes (UWV), Amsterdam, The Netherlands; 3 Research Center for Insurance Medicine, AMC-UMCG-VUmc-UWV, Amsterdam, The Netherlands

**Keywords:** Longitudinal cohort, Long-term sickness absence, Prediction models, Latent class analysis

## Abstract

*Purpose* Today, decreasing numbers of workers in Europe are employed in standard employment relationships. Temporary contracts and job insecurity have become more common. This study among workers without an employment contract aimed to (i) predict risk of long-term sickness absence and (ii) identify distinct subgroups of sick-listed workers. *Methods* 437 individuals without an employment contract who were granted a sickness absence benefit for at least two weeks were followed for 1 year. We used registration data and self-reported questionnaires on sociodemographics, work-related, health-related and psychosocial factors. Both were retrieved from the databases of the Dutch Social Security Institute and measured at the time of entry into the benefit. We used logistic regression analysis to identify individuals at risk of long-term sickness absence. Latent class analysis was used to identify homogenous subgroups of individuals. *Results* Almost one-third of the study population (n = 133; 30%) was still at sickness absence at 1-year follow-up. The final prediction model showed fair discrimination between individuals with and without long-term sickness absence (optimism adjusted AUC to correct for overfitting = 0.761). Four subgroups of individuals were identified based on predicted risk of long-term sickness absence, self-reported expectations about recovery and return to work, reason of sickness absence and coping skills. *Conclusion* The logistic regression model could be used to identify individuals at risk of long-term sickness absence. Identification of risk groups can aid professionals to offer tailored return to work interventions.

## Introduction

There is a positive association between work and one’s well-being, mental and physical health [1, 2]. In contrast, unemployment is strongly associated with poor health. The longer individuals are absent from work, the less likely they are to return [3–5]. Although long-term sickness absence makes up only a relatively small proportion of absences, it accounts for more than one-third of days off and 75% of sickness absence costs [6]. Early identification of individuals at risk of long-term sickness absence and an overview of factors associated with sickness absence duration can help occupational health professionals to target specific at-risk groups and identify effective early interventions to prevent long-term sickness absence [7]. Because occupational health services resources are limited, a differentiated approach is needed in occupational rehabilitation offering different levels of return to work support depending on individual characteristics and needs. Identification of groups of individuals, which are similar on certain characteristics, could be used as a triage tool to identify groups of claimants with the highest risk of long-term sickness absence and offer them suitable return to work interventions, based on the group characteristics.

Today, decreasing numbers of workers in Europe are employed in standard employment relationships. Temporary contracts and job insecurity have become more common [8]. Workers without a permanent employment contract, i.e. unemployed and temporary agency workers, represent a vulnerable group within the working population as they have poorer health status, and increased risk of long-term sickness absence and work disability [9, 10]. They have a greater distance to the labour market as they are characterised by lower credentials, lower income, more females, more (partly) disabled, and more immigrants [11]. The biopsychosocial model of illness and disability proposes that return to work of sick-listed workers depends on a combination of biological, psychological and social factors [12]. As not having a permanent employment contract has a negative impact on the development and maintenance of psychosocial health, the interaction between the factors of the biopsychosocial model is different between workers with and without a permanent employment contract [13]. Furthermore, the fact that workers without a permanent employment contract usually do not have a workplace to return to, might complicate their return to work process and prolong their sickness absence duration. In the Netherlands, this is reflected in a higher number of workers still being sick-listed at 1-year follow-up than workers with a permanent employment contract, and a higher inflow into work disability benefits after 2 years of sickness absence [14].

However, most studies on prognostic factors for long-term sickness absence focus on sick-listed employees, i.e. sick-listed workers with a permanent employment contract, rather than sick-listed workers without a permanent employment contract. Moreover, these studies focus on individuals with specific characteristics, for instance on individuals with specific diagnoses such as mental health problems [15–19], musculoskeletal disorders [20–23], or cancer [24, 25], or individuals belonging to a certain occupational group, such as healthcare workers [26]. These studies showed that sickness absence duration is mostly determined by factors that are not disorder-related. Although for occupational health professionals a prediction model that could be used for all diagnoses and occupational groups would be useful, such a model is currently missing.

In the present study, we included unemployed workers, temporary agency workers and workers with an expired fixed-term contract who received a sickness absence benefit for at least two weeks, covering all diagnoses and occupational groups. The aims of this study were to (i) predict sickness absence at 1-year follow-up and (ii) explore whether distinct subgroups of sick-listed workers could be identified, partly based on their predicted risk of long-term sickness absence.

## Methods

### Study Population

Dutch social security legislation allows sick-listed workers without a permanent employment contract to apply for a sickness absence benefit at the Dutch Social Security Institute (SSI; see text box) [27]. The study cohort included individuals who had been granted a sickness absence benefit by two regional offices of the Dutch Social Security Institute (SSI) between December 2016 and January 2017. All individuals were workers without a permanent employment contract, i.e. unemployed workers, temporary agency workers or workers with an expired fixed-term contract, sick-listed for at least two weeks. We excluded individuals who had been on sickness absence for less than two weeks as the probability to recover in this period is high, and therefore interventions for return to work are neither needed nor not cost-effective. In this study, we used a follow-up period of 1 year. We included all individuals still being sick-listed at the end of the 1-year follow-up period and all indivduals for whom the sickness absence benefit was ended because an individual had recovered. Individuals for whom the benefit was ended for other reasons, such as retirement, maternity leave or imprisonment, were excluded. The Medical Ethics Committee of Amsterdam UMC, VU University Medical Centre Amsterdam, gave ethical approval for this study and declared that no comprehensive ethical approval was needed.
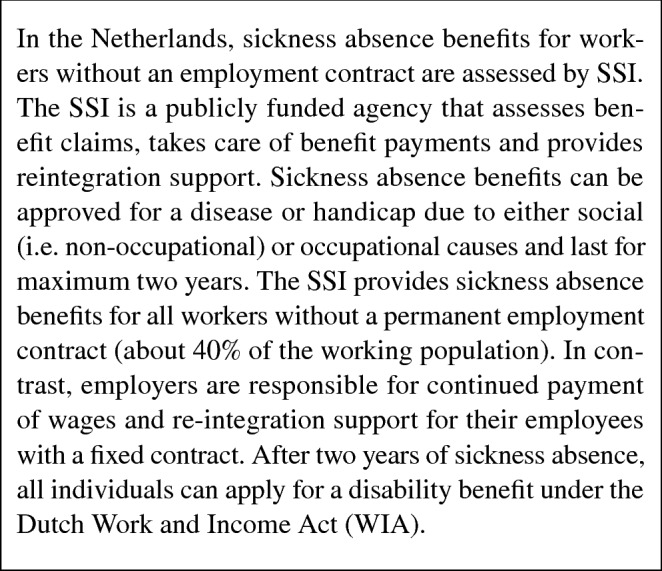


### Dependent Variable

The dependent variable, long-term sickness absence, was based on sickness absence duration data as registered by the SSI and dichotomized: individuals who had long-term sickness absence (i.e. still being sick-listed at 1-year follow-up) and individuals who did not have long-term sickness absence.

### Independent Variables

The aim of the prediction model was to identify, at the time of entry into the benefit, individuals who are at risk for sickness absence at 1-year follow-up. Hence, all independent variables were measured at baseline. Part of the independent variables were retrieved from the databases of the SSI: the socio-demographics age, gender, marital status, and educational level, as well as the work-related characteristics work status and occupational sector, and number of sickness absence days in the past year. In addition, a number of work-related, health-related and psychosocial characteristics were collected by the SSI using self-reported questionnaires that individuals needed to fill out when applying for the sickness absence benefit. Answering the self-reported questionnaires was part of the SSI process and thus obligatory. Work-related variables included self-reports on return to work expectations and possibilities to apply for jobs (yes/no). From the Dutch National Questionnaire Working Conditions (NEA) the following questions were used about the last job before sickness absence: labour conflict, physically demanding job, mentally demanding job, and work demands. The response categories were dichotomous for all questions: “mostly physical” and “mostly mental” for the last question, and “yes” and “no” for all other questions [28].

Health-related variables included reason of sickness absence (categorized as “mental disorders”, “musculoskeletal disorders”, and “other physical disorders”), expected duration of sickness absence (“less than 1 month”, “1–3 months”, “more than 3 months”, and ”don’t know”), and expected change in health during the next year (”improvement”, “deterioration”, and “no change”). General health condition was measured on a 5-point Likert scale ranging from “very bad” to “very good” [29]. Because only 37 individuals scored “very bad” on this question (< 4% of the total study population), we merged the categories “bad” and “very bad”.

Psychosocial factors were measured using the Well-Being Inventory (WBI) [30]. Individuals were asked whether they had problems with help-seeking, problem-solving, slowing down, ability to control events, whether they were worrying about the future in such extent that it prevented them from performing daily life activities, and whether they set high standards at work. The response options for all these variables were “yes” and “no”.

### Statistical analysis

Logistic regression analysis was used to determine prognostic factors to identify individuals with sickness absence at 1-year follow-up. The model was build using three steps. First, we performed univariable analyses to test the association of each independent variable with the outcome variable using likelihood ratio (LR) tests (cut-off score *p* > 0.15). Second, the variables remaining from the univariable analyses were tested for multicollinearity using variance inflation factors (VIFs). If VIF ≥ 10, the strongest predictor for long-term sickness absence was chosen [31]. Third, we selected the subset of predictors for the final model using a hybrid approach combining forward and backward selection procedures, adhering to Akaike’s Information Criteria as stopping rule [32].

Calibration, i.e. the agreement between observed and predicted risk of sickness absence, of the prediction model was assessed using the Hosmer–Lemeshow goodness-of-fit test. A *p *value ≥ 0.05 indicated that observed and predicted event rates were not significantly different. The disciminative ability of the model was evaluated using the area under the curve (AUC). The AUC is indicative of the precentage of correctly identified individuals at risk of long-term sickness absence. We interpreted AUC < 0.60 as failing, 0.60–0.69 as poor, 0.70–0.79 as fair, 0.80–0.89 as good, and 0.90–1.00 as perfect discrimination [33].

In general, prediction models perform better in the sample used to fit the model than in an external sample. To obtain a more accurate estimate of model performance, the internal validaty of the prediction model was examined by using a bootstrap approach [34]. We repeatedly drew 1000 samples from the study cohort, with replacement, and calculated the corrected AUC by comparing the prediction model in the bootstrap samples with the original sample [35].

Latent class analysis was used to identify homogenous, mutually exclusive subgroups (“clusters”) of sick-listed workers without an employment contract. Based on the predicted risk of sickness absence at 1-year follow-up, we calculated tertiles and divided the individuals into three risk groups: individuals with a low, medium and high predicted probability of long-term sickness absence. The latent class analysis was based on the predicted risk groups and the independent variables.

Latent class analyses were conducted specifying two to five clusters. We used Bayesian Information criterion (BIC) to assess model fit and determine the number of clusters in the optimal model [36, 37]. Individuals were assigned to the class with the highest posterior probability, i.e.to the class that best suited them. Average posterior class probabilities indicated the likelihood of class membership across all individuals whose maximum posterior probability was for that class and could be used to measure classification accuracy. The latent class analysis was considered accurate when the average posterior probabilities for all clusters were above 0.7 [36]. We interepreted the clusters based on the indicators with item-response probabilities of 0.7 or higher, as these indicators could be considered to be key characteristics of that cluster [38].

In general, all available variables can be used in latent class analysis. However, for practical purposes, selecting variables based on their usefulness for clustering was desirable as this improves interpretability of the model. Moreover, in the present study, most of the independent variables were retrieved from self-reported questionnaires, and shorter questionnaires are preferable in terms of costs and missing data. Therefore, we applied a variable selection approach based on the notion of BIC-based model selection [39]. Variables were sequentially considered for inclusion or exclusion from the set of variables selected for clustering based on their effect on BIC, maximized over the number of clusters and model parameterization.

All analyses were performed in RStudio for Windows, version 0.99.902.

## Results

The study population contained 437 individuals. Table 1 shows the baseline characteristics of the study population. The median sickness absence duration was 105 (Interquartile range [IQR] 46–396) days. After 1 year, 133 individuals (30%) were still on sickness absence.

**Table 1:** Descriptive statistics of the study population at baseline

	Study population N = 437	LTSA^a^ N = 133	Non-LTSA N = 304
Socio-demographics			
Age (years)	44.9 [12.3]^b^	45.3 [11.6]^b^	44.7 [12.6]^b^
Gender (female)	53%	62%	49%
Educational level^c^			
Low	34%	54%	25%
Secondary	39%	29%	43%
High	11%	14%	9%
Unknown	17%	4%	22%
Partner (yes)	65%	64%	66%
Work-related (characteristics of the previous job)			
Occupational sector			
Agriculture	11%	13%	11%
Finance	16%	17%	16%
Manufacturing	30%	26%	32%
Wholesale and retail	9%	11%	8%
Services	16%	22%	16%
Transportation	10%	7%	12%
Other	5%	5%	5%
Labour contract			
Unemployed workers	67%	73%	64%
Temporary agency workers	10%	7%	11%
Workers with an expired fixed-term contract	23%	20%	25%
Labour conflict (yes)	8%	12%	7%
Physically demanding job (yes)	59%	58%	60%
Mentally demanding job (yes)	46%	54%	43%
Work demands			
Mostly physical	63%	56%	65%
Mostly mental	37%	44%	35%
Return to work expectations (yes)	76%	67%	80%
Possibility to apply for jobs (yes)	34%	26%	38%
Health-related			
Reason of sickness absence			
Mental disorder	26%	32%	23%
Musculoskeletal disorder	40%	33%	43%
Other physical disorder	23%	18%	24%
Comorbidity of mental and physical disorders	12%	17%	10%
Number of sickness absence episodes in the past year	0.24 [0.52]^b^	0.24 [0.54]^b^	0.24 [0.52]^b^
Expected sickness absence duration			
Less than 1 month	12%	8%	13%
1–3 months	43%	28%	49%
More than 3 months	46%	65%	37%
General health condition			
(Very) bad	18%	23%	15%
Moderate	29%	35%	26%
Good	41%	36%	43%
Very good	13%	6%	16%
Expected health change			
No change	20%	23%	19%
Deterioration	8%	11%	7%
Improvement	72%	67%	74%
Limitations			
Difficulties with physical activities			
None	12%	6%	14%
Moderate	26%	27%	26%
Severe	62%	67%	60%
Difficulties with mental activities			
None	42%	33%	45%
Moderate	25%	23%	25%
Severe	34%	44%	29%
Relational or financial problems (yes)	24%	25%	24%
Psychosocial factors			
Help-seeking ability (yes)	55%	41%	61%
Worrying about the future (yes)	43%	50%	40%
Low control (yes)	53%	60%	50%
Problem-solving skills (yes)	64%	56%	67%
Set high standards at work (yes)	79%	84%	77%
Ability to slow down (yes)	27%	24%	29%

^a^*LTSA* long-term sickness absence, i.e. individuals still receiving sickness absence benefit at one-year follow-up

^b^Average and standard deviation

^c^Based on the highest level of education completed. Low = primary school, lower vocational education, lower secondary school. Secondary = intermediate vocational education, upper secondary school. High = upper vocational education, university

The final model predicting sickness absence at 1-year follow-up included three variables as predictors: educational level, expected sickness absence duration, and help-seeking ability. Table 2 shows the coefficients of the final prediction model. The *p *value of the Hosmer-Lemeshow goodness-of-fit test was 0.411, showing adequate calibration of the prediction model. The AUC of the final model was 0.777 (95% CI 0.731–0.822), showing fair discrimination for sickness absence at 1-year follow-up. Using bootstrap validation, the optimism-corrected AUC was 0.761 (95% CI 0.725–0.798). Multicollinearity was not assumed, as all VIF scores in the collinearity statistics for the multivariable model were < 10.

**Table 2:** Coefficients of the final model predicting sickness absence at 1-year follow-up

	OR [95% CI]	*p* value
Educational level		
Low	1	
Secondary	0.34 [0.21–0.58]	0.000
High	0.67 [0.33–1.39]	0.283
Unknown	0.10 [0.04–0.26]	0.000
Expected sickness absence duration		
Less than 1 month	1	
1–3 months	1.17 [0.51–2.69]	0.712
More than 3 months	2.82 [1.26–6.39]	0.012
Help-seeking ability (yes)	0.59 [0.37–0.94]	0.027

The best fitting model in the latent class analysis was the model with four clusters based on seven variables. Table 3 presents the characteristics of the four clusters that were named: sick-listed workers with positive expectations, sick-listed workers with mental limitations, sick-listed workers with physical limitations, and sick-listed workers with negative expectations. The cluster of sick-listed workers with positive expectations consisted mainly of individuals with a good general health condition, but with temporary musculoskeletal or other physical disorders. The majority of these individuals expected to recover within three months and fully return to work afterwards. Generally, individuals in the mental limitations cluster had mild and temporary mental disorders. The majority had positive expectations about return to work, but they expected longer episodes of sickness absence than individuals in the positive expectations cluster. Sick-listed workers with physical limitations suffered mostly from musculoskeletal or other physical disorders with a longer recovery time. They expected their recovery to be within one month to more than three months. Individuals with more severe mental disorders made up the largest of part of the cluster with negative expectations. They had a high risk of long-term sickness absence and negative coping skills.

**Table 3:** Characteristics of individuals in the four latent classes

	Positive expectations (n = 82) (%)	Mental limitations (n = 105) (%)	Physical limitations (n= 138) (%)	Negative expectations (n = 112) (%)
Risk of long-term sickness absence				
Low	67	42	31	2
Moderate	29	47	38	13
High	4	11	32	85
Return to work expectations (yes)	100	86	79	45
Reason of sickness absence				
Mental disorder	0	56	0	48
Musculoskeletal disorder	71	5	71	13
Other physical disorder	29	21	26	13
Comorbidity of mental and physical disorders	0	18	3	26
Expected sickness absence duration				
Less than 1 month	30	14	9	0
1–3 months	70	49	49	10
More than 3 months	0	37	43	90
Difficulties with mental activities				
None	78	2	75	12
Moderate	6	51	8	70
Severe	16	47	18	18
Help-seeking ability (yes)	85	59	69	10
Low control (yes)	15	83	28	84

There was a clear difference between the positive expectations cluster and the negative expectations cluster with respect to predicted risk of sickness absence and expected sickness absence duration: whereas all individuals in the positive expectations cluster expected to recover within 3 months, most individuals in the negative expectations cluster expected to be sick-listed for more than three months. Likewise, 67% of the individuals in the positive expectations cluster had a low risk of long-term sickness absence, while for 85% in the negative expectations cluster the model predicted a high risk. On the contrary, in the physical limitations cluster, both the expected sickness absence duration and the predicted risk of long-term sickness absence were much more varied. For sick-listed workers with negative expectations, the percentage with positive expectations about return to work was much lower (45%), than in the other three clusters. Concerning self-reported limitations and psychosocial factors, more than 75% of individuals in the positive expectations and physical limitations clusters reported no difficulties with mental activities and positive coping skills. In the mental limitations and negative expectations clusters, the majority reported moderate to severe difficulties with mental activities and negative coping skills. The average posterior probabilities of the four clusters were 0.79, 0.88, 0.86 and 0.89, respectively, indicating good classification accuracy.

## Discussion

The aims of this study were to (i) predict sickness absence in a vulnerable group of workers without an employment contract at 1-year follow-up, by building a model based on SSI registration data and self-reported questionnaires and (ii) explore whether distinct subgroups of sick-listed workers could be identified. The prediction model showed fair discrimination between individuals with and without long-term sickness absence based on three variables. Four types of sick-listed workers without an employment contract could be distinguished, partly based on the predicted risk of sickness absence at 1-year follow-up.

The prediction model for sickness absence at 1-year follow-up contained educational level, expected sickness absence duration, and help-seeking ability. The strongest predictor was self-reported expectations about sickness absence duration. This is in line with a previous study among sick-listed unemployed and temporary agency workers with psychological problems. That study reported that self-reported expectations about longer duration until full return to work was a strong prognostic factor for low work participation at long-term follow-up [17]. The other prognostic factor for long-term sickness absence in their final model was poor perceived health, which was not found to be a predictor in the present study. This could be due to the fact that in our model help-seeking ability was included, whereas their potential independent prognostic variables did not include psychosocial factors, or because their study population consisted only of workers with psychological problems which could have influenced perceived health.

Whereas only a few have studied prognostic factors in workers without a permanent employment contract, several studies have focused on prognostic factors for sickness absence duration for sick-listed employees. These studies showed that also for employees there is a relation between self-reported expectations and sickness absence duration. Among a Dutch cohort of sick-listed teachers, expectation of duration of sickness absence longer than three months was found to be a predictor of longer time until return to work [15]. Likewise, other studies have found a significant relation between self-reported expectations and return to work for injured employees and employees on sick leave for at least four weeks [40, 41]. A relation between psychosocial factors and sickness absence duration has been demonstrated as well [42–44]. Lower educational level proved to be predictive of long-term sickness absence in a Swedish cohort of individuals on sick leave for at least 55 days [45]. As previous studies demonstrated these relations among cohorts of employees with an employment contract, we have shown that these relation also hold for sick-listed individuals without an employment contract.

We found an optimism-corrected AUC of 0.733 (95% CI 0.707–0.758) for the model predicting sickness absence at 1-year follow-up. Previous studies on sickness absence duration for workers without a permanent employment contract did not report measures of the discriminative ability of the prediction models, thereby giving no information on the degree to which the predictions are valid for individuals from the underlying population [17]. Studies focusing on predicting sickness absence among employees did, and they found AUC values similar to our prediction model, i.e. ranging from 0.73 to 0.76 and showing fair discrimination between individuals with and without risk of long-term sickness absence [15, 23, 46]. However, most of these studies did not correct for over-optimism, and therefore their AUC values could be overestimated.

Four groups of sick-listed workers without an employment contract could be distinguished. Latent class analysis has previously been applied in occupational health studies concerning several populations, such as work disability for employees with diabetes and young adults with mental disorders [47, 48]. However, we are not aware of studies that applied latent class analysis to sick-listed employees without an employment contract. Latent class analysis is an effective method of data reduction and can guide stratified group-based intervention strategies. The results of the present study show that sick-listed workers in the negative expectations cluster, and possibly also individuals in the physical limitations cluster, are most in need of return to work support as they have the highest risk of long-term sickness absence. Return to work interventions for these workers could be tailored at the characteristics of the clusters. For instance, workers in the negative expectations cluster are characterized by low self-control and being sick-listed due to (comorbidity of physical and) mental disorders. They might benefit from an intervention developed for sick-listed unemployed workers with psychological problems, like supported employment and interventions aiming at goal-setting and increasing the sense of control [49, 50]. Most workers in the physical limitations cluster are sick-listed due to musculoskeletal disorders. They are more likely to benefit from other types of interventions, such as a participatory return to work program or an intervention aimed at examination, information, and recommendations to remain active [51, 52]. Contrary, for individuals in the positive expectations and mental limitations clusters minimal support to return to work may be sufficient as they have a low risk of long-term sickness absence.

## Strengths and Limitations

A strength of the present study is the heterogeneous study population. We included all workers without an employment contract who were granted a sickness absence benefit by two regional offices of the SSI. In contrast to most previous studies on longer term sickness absence that focused on individuals with specific diagnoses or individuals belonging to a certain occupational sector, our study population covered all diagnoses and occupational groups. As shown in previous studies, as well as in the prediction model of the present study, sickness absence duration is mostly determined by non-disorder related factors, and a prediction model that could be used for all diagnoses would be more useful for occupational healthcare professionals. Second, as answering self-reported questionnaires was part of the SSI working process and obligatory for all individuals, there is no non-response and thus no selection bias. Moreover, our study population consisted of unemployed workers, temporary agency workers and workers with an expired fixed-term contract as these are the most vulnerable group within the working population. This means that our results are of interest for social security agencies and occupational healthcare professionals. In addition, by using a variable selection algorithm for latent class analysis, we were able to find a parsimonious clustering. The clustering was partly based on self-reported questionnaires, and shorter questionnaires are preferred from a patient point of view. Moreover, as a parsimonious clustering is easier to interpret by occupational healthcare professionals, it better suits practical needs.

Identifying subgroups of individuals based on statistical methods helps to obtain an unbiased classification, i.e. to reduce the influence of professionals’ own values and judgements. However, a limitation of latent class analysis is that it could result in subgroups that are not recognizable by occupational healthcare professionals. A combined approach of statistical methods and group consensus could be used to ensure a validated and practically relevant classification. Another limitation of the study is that the self-reported questionnaires were developed for practical purposes. Questions were selected based on considerations of professionals in the field of sickness absence services and a literature search. This means that the questionnaires used by the SSI consisted of a set of single questions from several (validated) questionnaires. Moreover, it is possible that not all relevant predictors were measured.

## Practical Implications

The longer individuals are absent from work, the less likely they are to return to work [1]. Therefore, it is important for policymakers and occupational health professionals to know which factors predict long-term sickness absence. The present study showed that only three variables might be needed to fairly discriminate between individuals with and without long-term sickness absence. As asking only a limited number of variables takes less time, it is preferred in terms of user-friendliness.

Some individuals are more vulnerable to long-term sickness absence than others, especially individuals with a low educational level, negative expectations of sickness absence duration and lower help-seeking ability. As individuals can be expected to make a good estimation of the duration of their sickness absence themselves based on past experiences and personal and environmental factors, individuals who expect to recover in the short term may require less guidance from occupational healthcare professionals than individuals with negative recovery expectations [53, 54].

Because occupational health services resources are limited, a differentiated approach is needed in occupational rehabilitation. Sick-listed workers without an employment contract are a heterogeneous group consisting of several more homogenous subgroups. Some subgroups might benefit more from return to work interventions than others. Hence, the latent class analysis results could be used as a triage tool to identify groups of claimants with a high risk of long-term sickness absence, get insight into the characteristics of these groups, and offer each group return to work interventions tailored to their characteristics. The results of the present study indicate that return to work interventions should at least be offered to individuals belonging to the negative expectations cluster, and, in case of sufficient capacity of occupational health services, probably also to individuals in the physical limitations cluster. On the other hand, individuals belonging to subgroups with a low risk of long-term sickness absence (i.e. sick-listed workers in the positive expectation and mental limitations clusters) are likely to recover themselves within 1 year without extensive support from occupational healthcare professionals.

The predicted risk of long-term sickness absence and the partition of claimants into subgroups could be used by occupational health professionals at the start of the sickness absence period. It could be used as an additional source of information and guide professionals in selecting favourable return to work interventions for a particular claimant. During the rehabilitation process, new information might unfold and adjustment of the provided services might be needed. For instance, life events and differences in services or return to work interventions that sick listed workers receive might influence sickness absence duration. Hence, regular sickness absence monitoring is important to identify whether adjustment of return to work interventions might be beneficial.

## Concluding Remarks

This study showed that a logistic regression model could fairly discriminate between individuals with and without long-term sickness absence. Occupational healthcare professionals could use the outcome of the prediction model to identify individuals at risk of long-term sickness absence. The allocation of workers into distinct groups could be used for efficient allocation of return to work interventions tailored to the groups that will most benefit from it.
